# Supplementary material for: Drought stress induces salicylic acid accumulation, altering monoterpene profile and suppressing resin duct formation in Engelmann spruce
Source: PLoS One. 2026 May 14;21(5):e0349209. doi: 10.1371/journal.pone.0349209 (PMC13175483; doi:10.1371/journal.pone.0349209)
Supplement: S1 File — (DOCX) [file pone.0349209.s001.docx]

**Table S1**: The gradient used in the chromatographic separation of salicylic acid where the solvent. A: 10 mM ammonium acetate in water, solvent B: 10 mM ammonium acetate in acetonitrile.

| **Time, min** | **%A** | **%B** |
| --- | --- | --- |
| 0 | 95.0 | 5.0 |
| 0.10 | 95.0 | 5.0 |
| 4.00 | 50.0 | 50.0 |
| 4.10 | 0.0 | 100.0 |
| 5.00 | 0.0 | 100.0 |
| 5.10 | 95.0 | 5.0 |
| 7.00 | 95.0 | 5.0 |

**Table S2:** The hormone multiple reaction monitoring (MRM) transition quantifier ion (first transition listed) and qualifier ion (second transition listed), chromatographic retention time, cone voltage, and collision energy.

| **Hormone** | **Mass, g/mol** | **MRM Transition** | **Retention Time, min** | **Cone Voltage, V** | **Collision Energy, eV** |
| --- | --- | --- | --- | --- | --- |
| Salicylic acid | 138.1 | 137 > 65 | 0.7 | 50 | 24 |
|  |  | 137 > 93 | 0.7 | 50 | 14 |

**Table S3:** The temperature gradient used in the chromatographic separation of monoterpenes.

| **Time, min** | **Rate (°C min^-1^)** | **Target Value (°C)** | **Hold Time (min)** |
| --- | --- | --- | --- |
| 5.00 | 0.00 | 40.00 | 5.00 |
| 31.00 | 10.00 | 250.00 | 5.00 |
| 34.66 | 30.00 | 300.00 | 2.00 |

**Table S4:** The terpene transition quantifier ion (first transition listed) and qualifier ion (second transition listed) and chromatographic retention times.

| **Hormone** | **Mass, g/mol** | **Transition** | **Retention Time, min** |
| --- | --- | --- | --- |
| (+)-α-pinene | 136.2 | 93.1  93.1  92.1  77.1  79.1  121.1  105.1  94.1  80.1  67.1  136.2  107.1  53.1 | 9.45 |
| (-)-β-pinene | 136.2 | 93.1  91.1  69.1  79.1  77  92.1  94.1  80.1  121.1  67.1  136.2  53.1 | 10.441 |
| (+)-3-carene | 136.2 | 93.1  91.1  79.1  92.1  77.1  80.1  121.1  105.1  136.2  94.1  67.1  107.1 | 11.174 |
| camphene | 136.2 | 93.1  121.1  79.1  91.1  67.1  107.1  77.1  94.1  95.1  92.1  68.1  53.1 | 9.801 |
| β-phellandrene | 136.2 | 93.1  91.1  77  79.1  136.2  94.1  92.1  80.1  78.1  65.1  121.1  69.1 | 11.585 |
| sabinene | 136.2 | 93.1  91.1  77  79.1  92.1  94.1  136.1  80.1  69.1  78.1  121.1  65.1 | 10.37 |
| (+)-linalool | 154.3 | 71.1  93.1  55.1  69.1  41.1  43  80.1  67.1  91.1  121.1  92.1  79.1 | 12.914 |
| β-myrcene | 136.2 | 93.1  69.1  91.1  79.1  77.1  67.1  92.1  53.1  80.1  94.1  68.1  121.1 | 10.779 |
| γ-terpinene | 136.2 | 93.1  91.1  136.1  77  121.1  92.1  79.1  105.1  94.1  107.1  80.1  78.1 | 12.178 |
| terpinolene | 136.2 | 93.1  121.1  136.1  91.1  79.1  77  105.1  107.1  92.1  67.1  53.1  94.1 | 12.74 |

**Table S5:** Final concentrations of CA and CB salicylic acid standards when diluted with methanol.

| **C1-A (µg/mL)** | **C2-A**  **(µg/mL)** | **C3-A**  **(µg/mL)** | **C4-A**  **(µg/mL)** | **C5-A**  **(µg/mL)** | **C6-A**  **(µg/mL)** | **C7-A**  **(µg/mL)** | **C8-A**  **(µg/mL)** |
| --- | --- | --- | --- | --- | --- | --- | --- |
| 100.0 | 50.0 | 10.0 | 5.0 | 1.0 | 0.5 | 0.1 | 0.05 |
|  |  |  |  |  |  |  |  |
| **C1-B (µg/mL)** | **C2-B**  **(µg/mL)** | **C3-B**  **(µg/mL)** | **C4-B**  **(µg/mL)** | **C5-B**  **(µg/mL)** | **C6-B**  **(µg/mL)** | **C7-B**  **(µg/mL)** | **C8-B**  **(µg/mL)** |
| 120.0 | 60.0 | 12.0 | 6.0 | 1.2 | 0.6 | 0.12 | 0.06 |
