# Supplementary material for: Drought stress induces salicylic acid accumulation, altering monoterpene profile and suppressing resin duct formation in Engelmann spruce
Source: PLoS One. 2026 May 14;21(5):e0349209. doi: 10.1371/journal.pone.0349209 (PMC13175483; doi:10.1371/journal.pone.0349209)
Supplement: Attachment — Submitted filename: Response to reviewers.docx [file pone.0349209.s003.docx]

**Reviewers' comments:**

**Reviewer 1:**

**The most significant finding is the suppression of traumatic resin duct induction under chronic water stress conditions (Experiment 3). However, the manuscript should further explore the implications of the uncoupling of SA signaling observed between needles and stem phloem (Experiment 2).**

>We agree that this is an interesting angle, however, at the suggestion of R2 we have decided to refrain from extensively speculating on mechanisms that were beyond the scope of the present work. Accordingly, we have re-phrased this section to instead read “This could indicate that different environmental factors drive accumulation of SA in needle and stem tissues (Vanhatalo et al. 2015)…”, and no longer refer to a ‘decoupling’ as this was not directly tested or observed.

**The discussion should articulate the hypothesis that SA is modulating terpene biosynthesis as part of a protective response against drought-induced oxidative stress (ROS scavenging). Water stress leads to photosynthetic inhibition and the accumulation of reactive oxygen species (ROS).**

>We have added some text to address this idea, as suggested.

**The Methods section should be more detailed. The complete protocol for quantification of SA and monoterpenes (GC-MS/LC-MS specifications, column parameters, detection limits) should be fully described in the main body of the text, and not just in the captions or supplementary material.**

>We have included information in-text on the GC-MS temperature program. Column parameters and carrier gas flow rates are provided L231-239. Likewise, relevant instrument parameters for UPLC analyses are provided at L206-218. Additional detail on detection limits is now provided in the data acquisition section on L250-255.

We felt that detailed in-text discussion of solvent gradients and (Table S1) and especially listing of all monoterpene transition quantifier ions (Table S4) was simply too cumbersome to include in-text, but these details are transparently viewable in supplemental tables.

**Correct obvious typos (e.g., "forest instects" for "forest insects"; "steam water potential" for "stem water potential").**

>Corrected as suggested here.

**Acceptance for publication is conditional upon revision of the text to intensify the mechanistic discussion, especially regarding SA-JA antagonism and the antioxidant function of SA-induced monoterpenes.**

>We added relevant information along with citations, including a more detailed discussion of potential mechanistic roles of SA and monoterpene induction during drought stress. However, we have tried to be careful not to extend our discussion beyond the scope of what was observed and tested in the current studies as per the comments of R2 below. Accordingly, we now introduce ‘hormone crosstalk’ and ‘carbon starvation’ as potential competing hypotheses explaining a lack of resin duct formation in drought-stressed trees in response to MeJa.

**Reviewer 2:**

**Terms such as “apparency,” “resin duct density,” “basipetal signaling,” etc. should be briefly defined on first use for broader accessibility.**

>We now define ‘apparency’ (L425) at first use in the main text. The term ‘basipetal signaling’ could not be located in the text. ‘Resin duct density’ has been changed to ‘resin duct formation’ throughout to improve clarity.

**Some figures lack full clarity and could be expanded to briefly explain**

>Figure summaries have been added for each caption as per journal guidelines, which should help to improve the clarity of figures. We have also included some additional interpretation in figure captions where appropriate.

**Lack of explicit controls in Experiment 3**

>We are unclear on the meaning of ‘explicit controls’. Here the adjusted experimental variable is water level (n=2). It is already well established that application of MeJa induces traumatic resin ducts in spruces including the study species (e.g. Davis 2025, reference now included), so we did not consider it necessary to demonstrate that not applying MeJa does not result in resin duct production.

Reference:

Davis 2025. <https://doi.org/10.1007/s10886-025-01665-4>

**Using α = 0.10 for significance is unconventional. Must be justified or adjusted.**

>Several references have been added at this line to justify the use of 0.10 for interpreting statistical significance. This usage is increasingly common in ecological fields where variation can be extreme and study conditions may be impossible to identically replicate.

References:

Wasserstein et al. 2019: <https://doi.org/10.1080/00031305.2019.1583913>

Amrhein et al. 2019: <https://doi.org/10.1038/d41586-019-00857-9>

**Statistical assumptions (normality, variance homogeneity) are not mentioned. Without this, model validity is uncertain.**

>We have added a statement to this effect. We also now discuss the distribution of monoterpene data, and justify use of ANOVA from recent research.

Reference:

Blanca et al. 2017. <https://doi.org/10.7334/psicothema2016.383>

**ANOVA Model Structure Needs Detail. The phrase “watering treatment nested by time” is ambiguous.**

>This has been slightly revised to make it clearer that treatment effects are compared within each day, hence the term ‘nested within day’. To our knowledge, a ‘nested’ design is common statistical terminology (e.g., Schielzeth and Nakagawa 2012), and we are not sure how else to phrase this.

References:

Schielzeth and Nakagawa: <https://doi.org/10.1111/j.2041-210x.2012.00251.x>

**Effect Sizes Not Reported**

>For simple linear regressions or ANOVA models with only one factor (i.e., Figure 1), we now include coefficients of determination as an estimate of effect size.

For our nested two-way ANOVA model evaluating effects of SA concentration, watering treatment, and their interaction on monoterpene concentrations (Table 1), effect sizes can be generalized from the sum-of-squares column.

For our nominal logistic model (Figure 5), we now report the log-worth value for each model parameter in parenthetical statements as an estimate of parameter effect size.

**Randomization and blinding are not described. Greenhouse bench effects may exist.**

>Randomization and blind labeling are now described in the methods. All treatment positions were completely randomized on greenhouse benches.

**Some statements in the Discussion imply causal relationships not directly tested (e.g., SA antagonizing JA).**

>We agree, and refrain from stating this. Instead, we now refer to this as a possible explanation of why drought stress inhibited resin duct formation in response to MeJa, and provide other competing hypotheses as well (ie, carbon starvation).

**Conclusions should be rewritten to remain strictly within the boundaries of the data.**

>We have tried to be careful about this, while also providing some additional discussion as requested by R1.

**Language: The manuscript is mostly intelligible and readable, but improving grammar, phrasing, and clarity will enhance the professional quality of the work. A careful proofreading or light professional editing is recommended.**

>We have carefully proofread the ms and made edits as needed throughout to improve clarity where possible.
